# Improved Survival with Delayed Surgery at High-Volume Centers Versus Early Surgery at Low-Volume Centers for Pancreatic Cancer

**DOI:** 10.1245/s10434-025-19052-4

**Published:** 2026-01-15

**Authors:** Sara Sakowitz, Mampei Yamashita, McKensie Hammons, Timothy R. Donahue

**Affiliations:** 1 Division of Surgical Oncology, Department of Surgery, David Geffen School of Medicine, University of California Los Angeles, Los Angeles, CA USA; 2 Jonsson Comprehensive Cancer Center, David Geffen School of Medicine, University of California Los Angeles, Los Angeles, CA USA; 3 Department of Surgery, Massachusetts General Hospital, Boston, MA USA

**Keywords:** Pancreatic adenocarcinoma, Pancreatic cancer, Centralization of care, Leapfrog, Survival, High volume centers

## Abstract

**Background:**

Although the relationship between higher surgical volume and improved outcomes for pancreatic operations is well established, centralizing care to high-volume centers (HVC) may prolong the interval from diagnosis to surgery. This study sought to compare outcomes of patients who had longer wait times for upfront surgery for pancreatic cancer (PDAC) at HVCs with those of patients who underwent earlier surgery at low-volume centers (LVCs).

**Methods:**

Patients undergoing upfront pancreatic surgery for T1-3N0-2M0 PDAC were identified from the 2004–2023 National Cancer Database. High-volume centers were defined using Leapfrog criteria as centers performing ≥ 20 pancreatic resections/year, with others defined as LVCs. Patients who waited more than 28 days for resection at HVCs were classified as “long wait/high volume,” whereas those who underwent surgery in ≤ 14 days at LVCs were classified as “short wait/low volume.”

**Results:**

Among 15,310 patients meeting the inclusion criteria, 9598 (63%) were short wait/low volume and 5712 (37%) were long wait/high volume. In unadjusted analysis, long wait/high volume demonstrated superior 5-year survival (23% vs. 19%, *P* < 0.001, log-rank). After comprehensive risk adjustment, waiting for surgery at an HVC remained associated with reduced mortality hazard during 5 years of follow-up evaluation (hazard ratio [HR], 0.81; 95% CI, 0.77–0.85; *P* < 0.001). Considering acute endpoints, the long-wait/high-volume group demonstrated greater likelihood of complete (R0) resection and reduced 30-day mortality, but higher risk of nodal disease and upstaging at resection.

**Conclusion:**

Waiting for surgery at an HVC is associated with improved acute outcomes and superior overall survival compared with earlier operations at an LVC. These findings assuage concerns regarding the potential longer wait times for surgery associated with centralization of surgical care for PDAC.

**Supplementary Information:**

The online version contains supplementary material available at 10.1245/s10434-025-19052-4.

A lethal malignancy, pancreatic ductal adenocarcinoma (PDAC), is projected to become the second leading cause of cancer-related death by 2030.^1^ Surgery remains the only curative treatment for localized, non-metastatic disease. However, up to 30% of tumors are found to be unresectable at the time of diagnosis or surgical exploration.^2^ Given the aggressive tumor biology and rapid rate of progression considered a hallmark of the disease, many have advocated for expedited time to pancreatic resection from diagnosis.^3^

Paradoxically, however, in the contemporary era of oncologic care centralization, waiting times have broadly increased.^4^ Although a large body of evidence supports the volume-outcome relationship demonstrating that care at high-volume centers (HVCs) confers superior outcomes,^5^ these health systems face increasing caseloads yet finite numbers of surgeons and operating rooms (OR). As a result, patients referred to HVCs may experience delays of several weeks between diagnosis and surgery, a challenge less frequently encountered at low-volume centers (LVCs), at which scheduling may be more immediate but outcomes often inferior.

Importantly, waiting for pancreatic cancer resection has been associated with conflicting outcomes across the literature. Prolonged time to resection has been linked to increased tumor size,^6^ higher rates of palliative bypass,^7^ reduced likelihood of resection,^8,9^ and decreased overall survival.^10^ However, other international studies have found no significant impact of longer waiting duration on survival outcomes.^11–14^ Moreover, the potential mediating effect of hospital volume on the relationship of waiting time remains unclear. One study by Wu et al.^15^ using a Taiwanese cohort from 2004 to 2015 found improved outcomes at HVCs despite longer waiting times. However, differences in population characteristics and health system structure limit the generalizability of these findings to the modern U.S. setting. A clearer understanding of the impact of waiting for care at HVC could inform ongoing centralization efforts and guide interventions to improve care delivery.

In this national study of the American College of Surgeons Commission on Cancer-designated hospitals, we compared outcomes of patients experiencing longer wait times for pancreatic resection at HVCs with those of patients treated with expedited surgery at LVCs. We hypothesized that, compared with expedited surgery at LVCs, waiting for care at HVCs would be associated with improved short- and long-term oncologic outcomes.

## Methods

### Data Source

This was a retrospective study of the National Cancer Database (NCDB), the largest oncologic registry in the United States. Data quality is maintained by the American Cancer Society and the Commission on Cancer of the American College of Surgeons. The NCDB and participating hospitals are the source of the de-identified data and are used in this report. They have not verified and are not responsible for the statistical validity of the data analysis or the conclusions derived by the authors. The Institutional Review Board of the University of California, Los Angeles deemed this study exempt from full review due to the deidentified nature of the NCDB (IRB no. 24-5895).

### Study Population

**Fig. 1:**
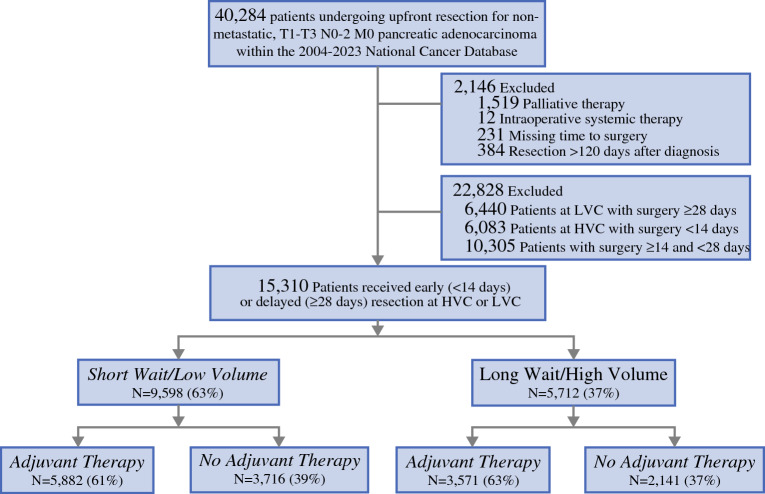
Study CONSORT diagram. Among patients referred for upfront surgical resection from 2004 to 2023, 15,310 met the initial study criteria. The patients then were stratified by both time to surgery and care at a high-volume center (HVC) in accordance with Leapfrog criteria ( ≥ 20 pancreatic resections/year). Patients receiving timely surgery (in  < 14 days) who were treated at low-volume, non-Leapfrog centers (LVCs), were categorized as the short-wait/low-volume group. Meanwhile, those receiving delayed surgery (in ≥ 28 days) who received care at high-volume, Leapfrog-designated programs, were considered the long-wait/high-volume cohort

All adults (age ≥ 18 years) with T1-3N0-2M0 PDAC diagnosed and histologically confirmed from 2004 to 2023 were tabulated from the NCDB using the *International Classification of Diseases for Oncology* third-edition codes. Staging was performed according to the eighth edition of the tumor-node-metastasis (TNM) staging system.^16^ Only those who received upfront surgical resection, including partial/total pancreatectomy with or without gastrectomy or duodenectomy, Whipple, or extended pancreatoduodenectomy, were considered. The study excluded patients who had clinical metastasis at diagnosis (*n* = 421) or had received intraoperative systemic therapy (*n* = 12). We also eliminated records that were missing the duration of time from diagnosis to surgery (*n* = 231) or that entailed surgery more than 120 days after diagnosis (*n* = 384) (Fig. 1).

### Variable Definitions

The NCDB data dictionary was used to define patient, disease, and hospital characteristics. Clinical and pathologic staging was ascertained using TNM criteria, eighth edition. The modified Charlson-Deyo Index was used to quantify the burden of chronic illness. Postsurgical margins are detailed as reported in the NCDB, with complete (R0) resection delineated as all margins, both grossly and microscopically, negative. In accordance with Leapfrog criteria, centers performing ≥ 20 pancreatic resections/year were designated HVC, with all the others considered LVC.

### Cohort Stratification

The median duration of time from diagnosis to resection was 20 days. We then defined early surgery as ≤ 14 days and delayed surgery as > 28 days from diagnosis to resection. Patients who experienced delayed resection at an HVC were grouped as the “long-wait/high-volume” cohort, whereas those who underwent early surgery at an LVC were considered the “short-wait/low-volume” group.

### Statistical Analysis and Study Outcomes

Normally distributed continuous data are presented as means with standard deviations (SDs), whereas non-normally distributed variables are reported as medians with interquartile ranges (IQRs). Categorical data are reported as group frequency (%). The significance of intergroup differences was evaluated using standard statistical tests.

Unadjusted survival was evaluated using the Kaplan-Meier method. Adjusted survival was considered using Cox proportional hazard models. We also developed logistic or linear regression models to evaluate the likelihood of certain secondary outcomes, described later.

To optimize model generalizability and minimize bias, all covariates were automatically selected for model inclusion using elastic net regularization.^17^ Models ultimately were adjusted for patient age, sex, comorbidity burden, sociodemographic characteristics, tumor size, clinical nodal stage, surgical approach, tumor location within the pancreas, and receipt of adjuvant chemotherapy, as well as for year of diagnosis, hospital region, and academic hospital status.

The primary study endpoint was overall survival during 5 years after diagnosis. To address immortal time bias, we defined survival as the period from resection to last contact or death. Patients alive at the last follow-up visit were censored.

After our main analysis, we performed several rigorous sensitivity analyses. First, to account for patient-clustering across institutions, we developed a mixed-effects Cox model based on a gamma frailty distribution, with patient factors comprising the first level and hospital-level effects the second level.^18^ Second, we separately investigated outcomes, stratifying them by tumor location. Third, we assessed survival among the subgroup of patients who received adjuvant chemotherapy. We performed a landmark analysis and evaluated survival endpoints among patients who survived the first 90 days after surgery as well as the first year after diagnosis. We assessed several secondary outcomes, including receipt of R0 resection, adequate lymphadenectomy ( ≥ 12 nodes harvested), positive nodes, upstaging at diagnosis, and duration of postoperative hospitalization, as well as factors linked with receipt of delayed resection at HVCs. Finally, to better distinguish the relative impact of surgical timing versus care at HVCs, we evaluated the counterfactual scenario of our analysis by comparing outcomes of early surgery at HVCs versus delayed management at LVCs.

Cox model outputs are detailed as hazard ratios (HRs) with 95% confidence intervals (CIs). Outputs of logistic or linear models are reported as adjusted odds ratios (AORs) or *β*-coefficients, respectively. Statistical significance was defined as an alpha of 0.05. All statistical analyses were performed using Stata 18.0 (StataCorp, College Station, TX, USA).

## Results

### Population Trends

**Fig. 2:**
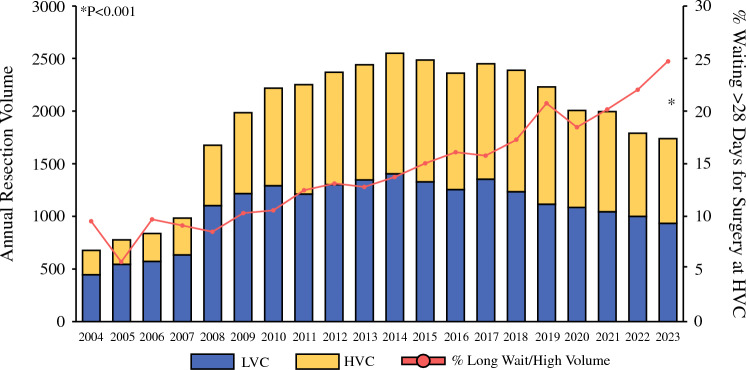
Trends in treatment. In accordance with Leapfrog criteria, high-volume centers (HVCs) were defined as those performing ≥ 20 resections per year (others were low-volume centers [LVCs]). Across the study period, the proportion of patients treated at HVCs increased, from 34% in 2004 to 46% in 2023 (*P* < 0.001 for trend). Furthermore, the percentage of patients waiting longer than 28 days for resection at HVCs grew from 10% in 2004 to 25% in 2023 (*P* < 0.001 for trend)

Across the study period, the proportion of patients who underwent upfront resection at HVCs increased from 34% in 2004 to 46% in 2023 (*P* < 0.001 for trend). Similarly, the percentage of patients waiting longer than 28 days for surgery rose from 20% in 2004 to 53% in 2023 (*P* < 0.001 for trend). The proportion of patients waiting longer than 28 days for surgery at HVCs increased from 10% in 2004 to 25% in 2023 (*P* < 0.001 for trend; Fig. 2).

### Cohort Characteristics

Of 15,310 patients meeting the study inclusion criteria, 9598 (63%) patients comprised the short-wait/low-volume group, whereas 5712 (37%) were classified as long wait/high volume. The median waiting time was 1 day for short wait/low volume and 41 days for long wait/high volume.

Comprehensive cohort characteristics are reported in Table 1. On the average, the long-wait/high-volume group was older (median, 71 vs. 67 years; *P* < 0.001) and had a higher comorbidity burden than the short-wait/low-volume group. Tumor size was clinically similar between the groups, but the long-wait/high-volume group more commonly presented with N2 disease (30% vs. 23%; *P* < 0.001). However, the proportions of patients who underwent pancreaticoduodenectomy were similar. Moreover, the patients in the long-wait/high-volume group traveled farther for care (median, 28.2 vs. 10.8 miles; *P* < 0.001), but less often received adjuvant radiotherapy (13% vs. 25%; *P* < 0.001).

**Table 1:** Demographic, clinical, and hospital characteristics^a^

	Short wait/low volume *n* (%)	Long wait/high volume *n* (%)	*P* Value
Patient	9598 (63)	5712 (37)	–
Hospital	772 (90)	89 (10)	–
Patient characteristics			
Median age: years (IQR)	67 (59–74)	71 (63–77)	< 0.001
Female sex	4833 (50)	2823 (49)	0.27
Median distance traveled: miles (IQR)	10.8 (4.9–27.1)	28.2 (11.0–69.3)	< 0.001
CDI			< 0.001
0–1	8620 (90)	4844 (85)	
2–3	978 (10)	868 (15)	
Mean tumor size (cm)	3.5 ± 2.5	3.5 ± 2.6	0.03
AJCC stage			< 0.001
IA	766 (8)	560 (10)	
IB	1475 (15)	806 (14)	
IIA	1035 (11)	416 (7)	
IIB	3103 (32)	1273 (22)	
III	3219 (34)	2657 (47)	
Clinical T stage			< 0.001
T1	1636 (18)	1334 (24)	
T2	4184 (45)	2755 (49)	
T3	3443 (37)	1514 (27)	
Clinical N stage			< 0.001
N0	4252 (45)	2694 (48)	
N1	3006 (32)	1240 (22)	
N2	2196 (23)	1656 (30)	
Tumor location			< 0.001
Head/neck	6803 (71)	3794 (66)	
Body	616 (6)	638 (11)	
Tail	1243 (13)	853 (15)	
Duct	72 (1)	20 (<1)	
NOS/overlapping	864 (9)	407 (7)	
Surgical approach			< 0.001
Partial/distal pancreatectomy	1520 (16)	1175 (21)	
Pancreatectomy/duodenectomy	6635 (69)	3920 (69)	
Total pancreatectomy	1290 (13)	565 (10)	
Pancreatectomy NOS	153 (2)	52 (1)	
Race			0.001
White	7942 (83)	4851 (85)	
Black	1103 (11)	577 (10)	
Asian/Pacific Islander	351 (4)	157 (3)	
Other	202 (2)	127 (2)	
Income percentile (%)			< 0.001
> 75	3112 (37)	2003 (40)	
51–75	2156 (25)	1226 (25)	
26–50	1792 (21)	1009 (20)	
0–25	1459 (17)	730 (15)	
Insurance coverage			< 0.001
Private	3222 (34)	1465 (26)	
Medicare	5241 (55)	3785 (67)	
Medicaid	582 (6)	242 (4)	
Not insured	309 (3)	57 (1)	
Other payer	105 (1)	103 (2)	
Adjuvant treatment			
Chemotherapy	5882 (62)	3571 (63)	0.13
Radiation	2407 (25)	739 (13)	< 0.001
Hospital factors			
Median annual caseload (IQR)	8 (4–13)	34 (25–54)	< 0.001
Hospital region			< 0.001
Northeast	1759 (18)	1442 (25)	
Midwest	2228 (23)	1474 (26)	
South	3881 (41)	2044 (36)	
West	1665 (17)	720 (13)	
Hospital type			< 0.001
Academic	3615 (38)	4280 (75)	
Community	3647 (38)	644 (11)	
Integrated network	2271 (24)	756 (13)	
Oncologic factors			
Median nodes examined (IQR)	13 (8–20)	19 (14–26)	< 0.001
Adequate lymphadenectomy	5615 (59)	4795 (84)	< 0.001
Margin status			< 0.001
No residual tumor	7240 (76)	4605 (81)	
Microscopic	1243 (13)	650 (11)	
Macroscopic	89 (1)	33 (1)	
Residual tumor NOS	919 (10)	385 (7)	
Hospitalization endpoints			
Median postoperative duration of hospitalization: days (IQR)	9 (6–14)	7 (5–10)	< 0.001
Readmission within 30 days	828 (9)	488 (9)	0.86
Acute mortality			
Within 30 days	457 (5)	108 (2)	< 0.001
Within 90 days	856 (9)	259 (5)	< 0.001

IQR, interquartile range; CDI, Charlson-Deyo Index; AJCC, American joint committee on cancer; NOS, not otherwise specified

^a^Reported as proportions unless otherwise noted. The hospital *n* (%) corresponds to the 2023 calendar year. Statistical significance was set at an alpha of 0.05.

### Survival After Resection

**Fig. 3:**
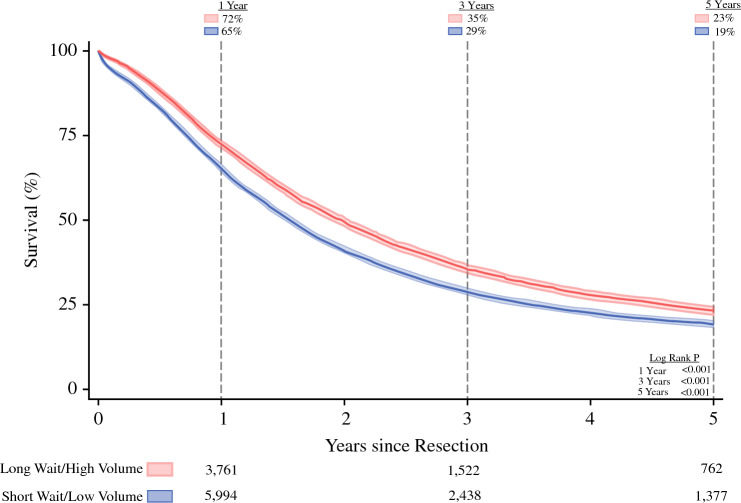
Overall survival. Kaplan-Meier time-to-event analysis of overall survival, with 95% confidence intervals. Patients experiencing longer waiting times to receive treatment at high-volume centers (long-wait/high-volume group) demonstrated better survival during 5 years after resection than those who faced a shorter wait to undergo resection at low-volume centers (short-wait/low-volume group). The at-risk numbers below the figure demonstrate the actual number of patients at risk

The median overall follow-up time was 1.6 years (IQR, 0.8–3.2 years). In unadjusted analyses, the long-wait/high-volume group demonstrated superior survival at 1 year (72% vs. 65%; *P* < 0.001, log-rank), 3 years (35% vs. 29%; *P* < 0.001, log-rank), and 5 years (23% vs. 19%; *P* < 0.001, log-rank) (Fig. 3).

After comprehensive risk adjustment, the long-wait/high-volume group continued to show a reduced hazard of mortality at 1 year (HR, 0.71; 95% CI, 0.66–0.77; *P* < 0.001), 3 years (HR, 0.79; 95% CI, 0.75–0.83; *P* < 0.001), and 5 years (HR, 0.81; 95% CI, 0.77–0.85; *P* < 0.001) (Table S1).

This effect remained true in a sensitivity analysis excluding deaths within 90 days after resection. Namely, delayed surgery at HVCs was still linked with reduced mortality at 1 year (HR, 0.81; 95% CI, 0.75–0.88; *P* < 0.001), 3 years (HR, 0.85; 95% CI, 0.80–0.89; *P* < 0.001), and 5 years (HR, 0.86; 95% CI, 0.82–0.91; *P* < 0.001).

### Multilevel Modeling

Multi-level modeling accounting for patient-clustering across hospitals yielded similar findings. Waiting longer than 28 days for resection at HVCs remained associated with reduced mortality hazard at 1 year (HR, 0.83; 95% CI, 0.75–0.90; *P* < 0.001), 3 years (HR, 0.91; 95% CI, 0.85–0.96; *P* < 0.001), and 5 years (HR, 0.89; 95% CI, 0.84–0.94; *P* < 0.001).

### Subgroup Analysis Stratifying by Tumor Location

**Fig. 4:**
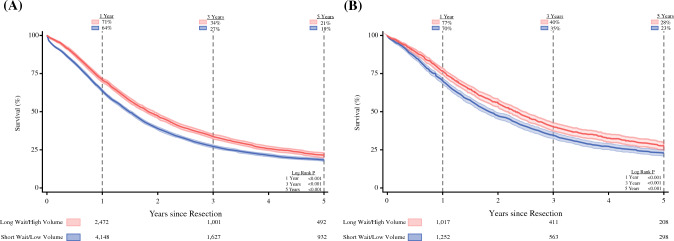
Overall survival stratified by tumor location. Kaplan-Meier time-to-event analysis evaluating the overall survival of patients who experienced a long wait for care at high-volume centers (long-wait/high-volume group) versus those who received early treatment at low-volume centers (short-wait/low-volume group), with 95 % confidence intervals*.* Among the patients with lesions in the **A** pancreatic head or **B** body/tail, the long-wait/high-volume group demonstrated better survival during 5 years after resection than the short-wait/low-volume group. The at-risk numbers detail the actual number of patients at risk

Among the 10,597 patients with pancreatic head tumors, 3794 (36%) received delayed care at HVCs, whereas 6803 (64%) underwent early surgery at LVCs. The patients in the long-wait/high-volume group demonstrated superior unadjusted survival at 1 year (71% vs. 64%; *P* < 0.001, log-rank), 3 years (34% vs. 27%; *P* < 0.001, log-rank), and 5 years (21% vs. 18%; *P* < 0.001, log-rank) (Fig. 4A). After risk adjustment, these differences persisted at 1 year (HR, 0.70; 95% CI, 0.65–0.77; *P* < 0.001), 3 years (HR, 0.78; 95% CI, 0.74–0.83; *P* < 0.001), and 5 years (HR, 0.81; 95% CI, 0.76–0.85; *P* < 0.001).

Among the 3350 patients with body or tail tumors, 1491 (45%) waited for care at HVCs. The long-wait/high-volume group again showed superior unadjusted survival at 1 year (77% vs. 70%; *P* < 0.001, log-rank), 3 years (40% vs. 35%; *P* < 0.001, log-rank), and 5 years (28% vs. 23%; *P* < 0.001, log-rank) (Fig. 4B). After adjustment, survival remained higher in this group at 1 year (HR, 0.73; 95% CI, 0.62–0.87; *P* < 0.001), 3 years (HR, 0.84; 95% CI, 0.75–0.94; *P* = 0.002), and 5 years (HR, 0.85; 95% CI, 0.76–0.94; *P* = 0.002).

### Subgroup Analysis of Patients Receiving Adjuvant Chemotherapy

**Fig. 5:**
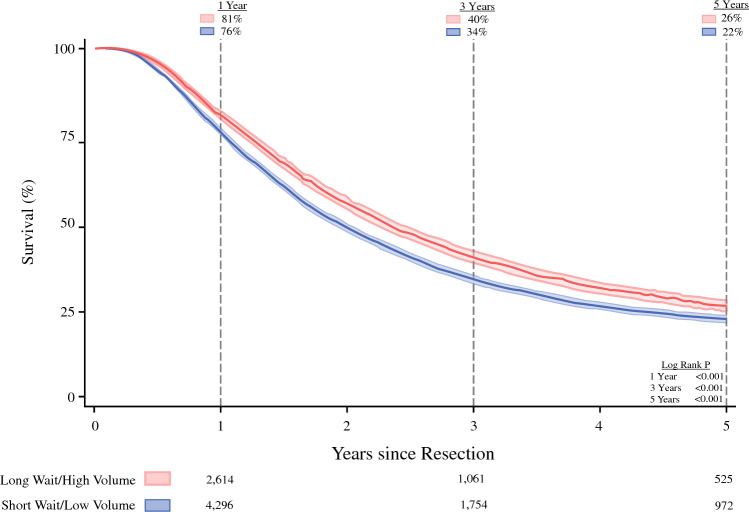
Overall survival among patients receiving adjuvant chemotherapy. Kaplan-Meier time-to-event analysis of overall survival among patients who received upfront surgical resection followed by adjuvant chemotherapy, with 95% confidence intervals. Patients waiting to receive care at high-volume centers (long-wait/high-volume group) demonstrated improved survival during 5 years after surgery compared with those receiving early resection at low-volume centers (short-wait/low-volume group). The at-risk numbers below the figure demonstrate the actual number of patients at risk

Of the 9453 patients who received adjuvant chemotherapy after surgery, 5882 (62%) were in the short-wait/low-volume group, and 3571 (38%) were in the long-wait/high-volume group. The patients in the latter group continued to demonstrate superior unadjusted survival outcomes at 1 year (81% vs. 76%; *P* < 0.001, log-rank), 3 years (40% vs. 34%; *P* < 0.001, log-rank), and 5 years (26% vs. 22%; *P* < 0.001, log-rank) (Fig. 5). After risk adjustment, delayed surgery at HVCs remained linked with reduced mortality hazard at 1 year (HR, 0.80; 95% CI, 0.72–0.90; *P* < 0.001), 3 years (HR, 0.84; 95% CI, 0.79–0.90; *P* < 0.001), and 5 years (HR, 0.86; 95% CI, 0.81–0.91; *P* < 0.001).

### Landmark Analysis of 1-Year Survivors

Among the 10,415 patients who survived the first year after diagnosis, 4187 (40%) had delayed surgery at HVCs. In unadjusted analysis, this group demonstrated superior survival at 3 years (49% vs. 44%; *P* < 0.001, log-rank) and 5 years (32% vs. 30%; *P* < 0.001, log-rank). After comprehensive adjustment, delayed surgery at HVCs remained associated with reduced mortality hazard at 3 years (HR, 0.87; 95% CI, 0.81–0.93; *P* < 0.001) and 5 years (HR, 0.89; 95% CI, 0.84–0.95; *P* < 0.001).

### Secondary Endpoints

**Fig. 6:**
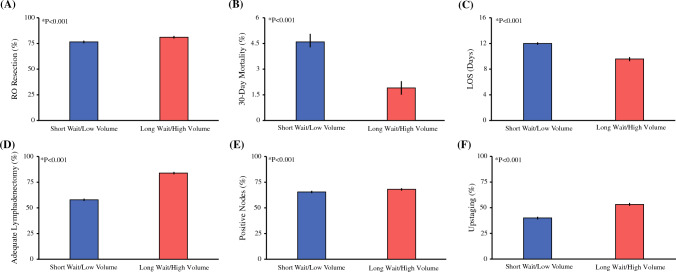
Secondary endpoints. After comprehensive risk adjustment for patient, disease, and hospital factors, patients experiencing a prolonged wait for pancreatic resection at high-volume centers (long-wait/high-volume group) demonstrated **A** greater adjusted risk of complete (R0) resection and **B** reduced likelihood of mortality within 30 days after surgery compared with those treated with early surgery at low-volume centers (short-wait/low-volume group). **C** Moreover, the long-wait/high-volume group experienced a 1.91-day reduction in postoperative length of stay (LOS). Considering oncologic endpoints, the long-wait/high-volume group experienced **D** higher risk-adjusted rates of adequate lymphadenectomy (≥12 nodes), but also faced **E** greater risk of positive nodes and **F** upstaging at surgical resection

After full adjustment, waiting for treatment at an HVC remained associated with a 24% increased likelihood of a complete (R0) resection (AOR, 1.24; 95% CI, 1.13–1.37; *P* < 0.001) and a 1.91-day shorter postoperative hospital stay (95% CI, –2.30 to –1.52 days; *P* < 0.001) (Table S2). The patients in the long-wait/high-volume group also had significantly greater odds of undergoing an adequate lymph node analysis (AOR, 3.39; 95% CI, 3.07–3.76; *P* < 0.001), but were more likely to experience pathologic upstaging at resection (AOR, 1.28; 95% CI, 1.16–1.40; *P* < 0.001). Finally, care at HVCs was associated with a significantly reduced risk of 30-day mortality (AOR, 0.36; 95% CI, 0.28–0.46; *P* < 0.001), but similar odds of 30-day readmission (AOR, 0.97; 95% CI, 0.85–1.12; *P* = 0.70) (Fig. 6).

### Counterfactual Analysis

We additionally evaluated the counterfactual scenario of our main analysis, comparing early surgery at HVCs with delayed care at LVCs. Receipt of early surgery at HVCs remained associated with superior survival at 1 year (HR, 0.78; 95% CI, 0.72–0.84; *P* < 0.001), 3 years (HR, 0.84; 95% CI, 0.80–0.89; *P *< 0.001), and 5 years (HR, 0.85; 95% CI, 0.80–0.89; *P* < 0.001).

#### Factors Linked With Delayed Care at HVCs

In the multivariate analysis, several factors were associated with receiving delayed pancreatic resection at an HVC compared to early surgery at an LVC. Older age (AOR, 1.03/year; 95% CI, 1.02–1.03/year; *P* < 0.001), greater comorbidity burden (AOR, 1.13/point; 95 % CI, 1.08–1.18/point; *P* < 0.001), increasing nodal stage (AOR, 1.20; 95% CI, 1.14-1.26; *P* < 0.001), and more recent year of diagnosis (AOR, 1.12/year; 95 % CI, 1.11–1.13/year; *P* < 0.001) all were linked with a higher likelihood of waiting for resection at an HVC. In contrast, Asian/Pacific Islander race (AOR, 0.78; 95% CI, 0.61–0.98; *P* = 0.03; reference: white), rural residence (AOR, 0.69; 95% CI, 0.50–0.95; *P* < 0.001; reference: metropolitan), lower educational attainment (0-25th percentile: AOR, 0.65; 95% CI, 0.56-0.77; *P* < 0.001); reference: 76-100th percentile), and lack of insurance (AOR, 0.44; 95% CI, 0.31–0.62; *P* < 0.001; reference: private insurance) were associated with lower odds of delayed surgery at an HVC.

## Discussion

The volume-outcome relationship has led to increasing centralization of PDAC care to HVCs.^19,20^ However, concerns remain about whether this shift, often resulting in longer waiting times for surgery, may negatively affect outcomes.^4^

In this national registry analysis, we assessed the intersection of hospital volume and time to surgery, finding that delayed resection at HVCs was associated with improved overall survival at 1, 3, and 5 years compared with expedited surgery at LVCs. Patients receiving delayed resection at HVCs had higher rates of R0 resection and lower 30-day mortality, although they more frequently presented with characteristics suggestive of disease progression at the time of surgery. These findings have important implications for the modern surgical management of PDAC.

Historically, prompt surgery was thought essential to avoid disease progression or dissemination in PDAC.^3^ Yet, evidence on the impact of waiting time for surgery has been mixed.^6,7,9,10,13,21,22^ Our findings offer reassurance that moderate delays because of referral to HVCs do not compromise outcomes.

Survival advantages at HVCs persisted across multiple analytical approaches, including comprehensive risk adjustment, hierarchical modeling to account for patient-clustering, and several rigorous subgroup analyses. Although the NCDB lacks granularity to parse the precise factors responsible for such findings, we proffer several potential contributors. Approximately 50% of patients who waited for care at HVCs were referred there, suggesting that these largely academic, regional referral centers may offer access to subspecialized surgeons, pathologists, and radiologists, as well as multidisciplinary tumor boards, advanced treatments or clinical trials, and expanded ancillary or supportive care services. Furthermore, cumulative surgeon and hospital experience not only may improve operative strategy and decision-making, but also may contribute to superior risk-stratification and early identification and management of postoperative complications.^23^ These multidimensional advantages may contribute to a smoother operative course and faster recovery, which may be of particular importance given the baseline high morbidity associated with pancreatic resection.^15^ Notably, our work suggests that these factors may more significantly shape patient outcomes than several weeks’ delay in surgical care.

Consistent with this, we found that patients undergoing delayed surgery at HVCs were more likely to undergo R0 resection and adequate lymphadenectomy, to have shorter hospital stays, and to experience lower 30-day mortality. Despite traveling greater distances, their 30-day readmission rates were similar to those of patients treated at LVCs. Interestingly, despite clinically similar rates of N1-N2 disease at presentation across cohorts, the long-wait/high-volume group also had higher rates of pathologic upstaging at resection and new nodal involvement on surgical pathology. Although this might initially suggest disease progression as found in prior investigations,^6,7^ these findings also may reflect differences in pathologic evaluation by specialized pathologists at HVCs. The latter interpretation is more consistent with improved survival outcomes observed and importance of high-quality pathologic assessment in oncologic surgery.

Finally, we observed that patients waiting for treatment at HVCs similarly often received adjuvant chemotherapy relative to those treated earlier at LVCs. Given the critical role of adjuvant therapy in treating systemic disease, this finding suggests that centralization of care may not negatively influence access to ongoing postoperative treatment. However, to reduce the travel distance patients face, these centers may benefit from expanding their networks in a “hub and spoke” model to facilitate high-quality surgical and adjuvant care while minimizing logistical burdens on patients.^24,25^

Critically, however, access to HVC care is not uniform. Rural residence, Medicaid insurance, and lack of insurance all were associated with lower odds of delayed surgery at HVCs, highlighting disparities in access to high-quality surgical care.^26–28^ Vulnerable patients may not recognize the benefits of referral to high-quality centers,^29^ and may lack transportation^30^ or support systems.^31^ Meanwhile, many (~70%) LVC patients received their diagnosis and treatment at the same institution. It is unclear whether these patients were offered referrals or simply scheduled for the next available surgery. In the setting of equipoise on timing^6,10,13,21,22^ but strong evidence supporting volume-based outcomes,^19,20,32^ efforts are needed to ensure equitable access to HVCs for all patients. Institutional and system-wide interventions should aim to identify and overcome barriers to referral for socioeconomically or geographically disadvantaged populations.

To further improve efficiency and reduce waiting times for surgery, several operational strategies at HVCs have shown promise. Centralized intake systems, such as those used in Canada and the Netherlands, prioritize operating room availability and streamline triage between academic and affiliate hospitals.^33,34^ Although not without limitations, this system has been implemented in at least one high-volume, tertiary center in the United States, with promising results.^35^ Internally, HVCs may benefit from operating room efficiency measures, including dedicated facilitators to manage scheduling and improve flow^36^ or data-driven block-scheduling to reduce bottlenecks.^37^ Expanding patient navigator services also may help reduce delays in preoperative workup and coordination. Altogether, these center-level efforts could have a profound impact on hospital operations and patient access to timely care.

Finally, and perhaps most notably, this analysis focused on patients undergoing upfront surgical resection to limit cohort heterogeneity and isolate the effect of surgical timing and hospital volume. The NCDB does not capture chemotherapy regimen, number of cycles, or sequencing relative to surgery, and therefore cannot distinguish patient-level reasons for treatment delays from hospital-level factors. However, accumulating evidence from several landmark trials supports neoadjuvant therapy for selected PDAC patients,^38–41^ and we anticipate that differences between HVCs and LVCs may widen in the contemporary multimodal era. Across cancer types, HVCs consistently demonstrate more comprehensive multidisciplinary evaluation, greater specialist availability,^42,43^ and higher delivery of multimodal therapy into clinical trial enrollment.^44,45^ Expanding access to HVCs, and improving care coordination between these centers and affiliated satellite sites will be essential to ensuring equitable and high-quality PDAC care. Future studies using more granular multi-institutional registries will be needed to evaluate whether patients who receive neoadjuvant multimodal therapy, more common at HVCs and often associated with additional treatment delays, experience outcomes similar to those of patients undergoing upfront surgery alone.

Our study had several limitations. We lacked data on symptom onset, reasons for treatment timing, and adjuvant therapy delivered outside reporting institutions. We could not assess patients who were intended for surgery but progressed before resection. Tumor resectability was inferred using clinical American Joint Committee on Cancer (AJCC) staging due to NCDB limitations. Moreover, in defining early surgical management as ≤ 14 days earlier and delayed care as > 28 days later, we aimed to construct a conceptual framework exploring one potential consequence of care centralization. Although a 14-day difference may not necessarily yield differential disease progression, this period may permit care coordination, surgical planning, and multidisciplinary team evaluation. Furthermore, we acknowledge that some delays may reflect preoperative optimization, which may have contributed to the superior outcomes observed in the HVC group. Rather than view this as a potential confounder, however, we considered preoperative optimization as a possible integral component of HVCs’ care algorithms. Our counterfactual analysis, comparing early surgery at HVC with delayed surgery at LVC, provided an additional test confirming that the associations between timing, hospital volume, and survival are not solely driven by preoperative management.

Our study was limited to overall survival. Future work should examine recurrence, disease-free survival, and patient-reported outcomes. Finally, moving beyond analyses of upfront surgery alone, future studies of more granular multi-institutional registries should adopt our analytical framework and consider the implications of delayed multimodal treatment onset at HVCs.

In conclusion, delayed surgery at HVCs was associated with improved survival longer than 5 years after diagnosis. Moreover, waiting for HVC care was linked with improved oncologic outcomes, including increased likelihood of R0 and adequate lymph node analysis. These findings support ongoing centralization of complex pancreatic cancer care and counter concerns that longer wait times for surgery at HVCs may harm patients. Instead, they suggest that the quality of surgical, pathologic, and multidisciplinary care at HVCs may more than compensate for modest delays in time to surgery.

## Supplementary Information

Below is the link to the electronic supplementary material.

Supplementary file1 (DOCX 32 KB)
