# Supplementary material for: Improved Survival with Delayed Surgery at High-Volume Centers Versus Early Surgery at Low-Volume Centers for Pancreatic Cancer
Source: Ann Surg Oncol. 2026 Jan 15;33(5):4049–60. doi: 10.1245/s10434-025-19052-4 (PMC13083458; doi:10.1245/s10434-025-19052-4)
Supplement: Supplementary file 1 — Supplementary file1 (DOCX 32 KB) [file 10434_2025_19052_MOESM1_ESM.docx]

**SUPPLEMENTARY APPENDIX**

**Improved Survival with Delayed Surgery at High Volume Centers versus Early Surgery at Low Volume Centers for Pancreatic Cancer**

Sara Sakowitz MD MPH MBA^1,2,3^, Mampei Yamashita MD^1^, McKensie Hammons BS^1^, Timothy R. Donahue MD^1,2^

^1^ Division of Surgical Oncology, Department of Surgery, David Geffen School of Medicine, University of California Los Angeles, Los Angeles, CA.

^2^Jonsson Comprehensive Cancer Center, David Geffen School of Medicine, University of California Los Angeles, Los Angeles, CA, USA.

^3^Department of Surgery, Massachusetts General Hospital, Boston, MA.

**Corresponding Author:**

Timothy R. Donahue MD [tdonahue@mednet.ucla.edu](mailto:tdonahue@mednet.ucla.edu)

**Contents**

[Supplemental Table S1: Cox Regression Model Output for Five Year Survival 3](#_Toc216352844)

[Supplemental Table S2: Acute Oncologic & Five Year Survival Outcomes. 5](#_Toc216352845)

# **Supplemental Table S1: Cox Regression Model Output for Five Year Survival**

Output of Cox proportional hazards model for survival over five years. High Volume Centers (HVC) were defined using Leapfrog criteria as performing ≥20 pancreatic resections/year, respectively (others: LVC). Surgery >28 days following diagnosis was defined as delayed, while resection ≤14 days from diagnosis was considered early.

*HVC, High Volume Center; LVC, Low Volume Center; NOS, Not otherwise specified

| **Factor** | **Hazard Ratio** | **Confidence Interval** | **P-Value** |
| --- | --- | --- | --- |
| Delayed Surgery at HVC  (Reference: Early Surgery at LVC) | 0.81 | 0.77-0.85 | <0.001 |
| Age at Diagnosis (Per Year) | 1.01 | 1.01-1.01 | <0.001 |
| Female Sex (Reference: Male) | 0.95 | 0.91-0.99 | 0.02 |
| Charlson-Deyo Index ≥2 (Reference: <2) | 1.25 | 1.17-1.33 | <0.001 |
| Increasing Year of Diagnosis (Reference: 2004) | 0.98 | 0.98-0.99 | <0.001 |
| Receipt of Adjuvant Chemotherapy | 0.61 | 0.58-0.64 | <0.001 |
| *Race* |  |  |  |
| White | Reference | - | - |
| Black | 0.97 | 0.91-1.04 | 0.45 |
| Asian/Pacific Islander | 0.92 | 0.82-1.04 | 0.20 |
| Other | 0.95 | 0.81-1.11 | 0.49 |
| *Income Percentile* |  |  |  |
| >75% | Reference | - | - |
| 51-75% | 1.17 | 1.10-1.23 | <0.001 |
| 26-50% | 1.14 | 1.08-1.21 | <0.001 |
| 0-25% | 1.11 | 1.04-1.19 | 0.003 |
| *Rural/Urban Status* |  |  |  |
| Metropolitan | Reference | - | - |
| Urban | 1.04 | 0.98-1.11 | 0.23 |
| Rural | 1.05 | 0.89-1.23 | 0.55 |
| Nodal Stage (Per Increase in N Stage) | 1.39 | 1.35-1.42 | <0.001 |
| Tumor Stage (Per Increase in T Stage) | 1.14 | 1.11-1.18 | <0.001 |
| *Surgical Approach* |  |  |  |
| Partial/distal pancreatectomy | Reference | - | - |
| Pancreatectomy/duodenectomy | 1.12 | 1.03-1.21 | 0.007 |
| Total pancreatectomy | 1.16 | 1.06-1.28 | 0.001 |
| Pancreatectomy NOS | 1.05 | 0.87-1.26 | 0.63 |
| *Tumor Location* |  |  |  |
| Head/Neck | Reference | - | - |
| Body | 0.94 | 0.86-1.04 | 0.22 |
| Tail | 0.99 | 0.91-1.08 | 0.81 |
| Duct | 0.85 | 0.65-1.11 | 0.23 |
| NOS/Overlapping | 0.99 | 0.91-1.07 | 0.72 |
| *Hospital Region* |  |  |  |
| Northeast | Reference | - | - |
| Midwest | 1.22 | 1.15-1.31 | <0.001 |
| South | 1.16 | 1.10-1.23 | <0.001 |
| West | 1.07 | 1.00-1.15 | 0.06 |

# **Supplemental Table S2: Acute Oncologic & Five Year Survival Outcomes.**

Following comprehensive risk-adjustment, waiting for care at High Volume Centers (HVC) remained associated with improved oncologic and survival outcomes, relative to treatment at Low Volume Centers. Outcomes are reported as Adjusted Odds Ratios (AOR), unless otherwise indicated, with 95% Confidence Intervals (CI) and P-Values. Statistical significance was considered α=0.05.

|  | ***Adjusted Odds Ratio*** | ***95% CI*** | ***P-Value*** |
| --- | --- | --- | --- |
| **Margin-Negative Resection (R0)** | | | |
| *Short Wait/Low Volume* | Ref | - | - |
| *Long Wait/High Volume* | 1.24 | 1.13-1.37 | <0.001 |
| **Adequate Lymphadenectomy (≥12 nodes resected)** | | | |
| *Short Wait/Low Volume* | Ref | - | - |
| *Long Wait/High Volume* | 3.39 | 3.07-3.76 | <0.001 |
| **Upstaging at Resection** | | | |
| *Short Wait/Low Volume* | Ref | - | - |
| *Long Wait/High Volume* | 1.28 | 1.16-1.40 | <0.001 |
| **Duration of Hospitalization (β)** | | | |
| *Short Wait/Low Volume* | Ref | - | - |
| *Long Wait/High Volume* | -1.91 | -2.30, -1.51 | <0.001 |
| **30-Day Mortality** | | | |
| *Short Wait/Low Volume* | Ref | - | - |
| *Long Wait/High Volume* | 0.36 | 0.28-0.46 | <0.001 |
| **One Year Survival (HR)** | | | |
| *Short Wait/Low Volume* | Ref | - | - |
| *Long Wait/High Volume* | 0.71 | 0.66-0.77 | <0.001 |
| **Three Year Survival (HR)** | | | |
| *Short Wait/Low Volume* | Ref | - | - |
| *Long Wait/High Volume* | 0.79 | 0.75-0.83 | <0.001 |
| **Five Year Survival (HR)** | | | |
| *Short Wait/Low Volume* | Ref | - | - |
| *Long Wait/High Volume* | 0.81 | 0.77-0.85 | <0.001 |
